# Epigallocatechin‐3‐*O*‐gallate up‐regulates microRNA‐199a‐3p expression by down‐regulating the expression of cyclooxygenase‐2 in stimulated human osteoarthritis chondrocytes

**DOI:** 10.1111/jcmm.12897

**Published:** 2016-08-12

**Authors:** Zafar Rasheed, Naila Rasheed, Hani A. Al‐Shobaili

**Affiliations:** ^1^ Department of Medical BiochemistryCollege of MedicineQassim UniversityBuraidahSaudi Arabia; ^2^ Department of DermatologyCollege of MedicineQassim UniversityBuraidahSaudi Arabia

**Keywords:** osteoarthritis, chondrocytes, EGCG, hsa‐miR‐199a‐3p, COX‐2

## Abstract

Osteoarthritis (OA) is a most common form of arthritis worldwide leading to significant disability. MicroRNAs (miRNAs) are non‐coding RNAs involved in various aspects of cartilage development, homoeostasis and pathology. Several miRNAs have been identified which have shown to regulate expression of target genes relevant to OA pathogenesis such as matrix metalloproteinase (MMP)‐13, cyclooxygenase (COX)‐2, *etc*. Epigallocatechin‐3‐*O*‐gallate (EGCG), the most abundant and active polyphenol in green tea, has been reported to have anti‐arthritic effects, however, the role of EGCG in the regulation of miRNAs has not been investigated in OA. Here, we showed that EGCG inhibits COX‐2 mRNA/protein expression or prostaglandin E_2_ (PGE_2_) production *via* up‐regulating microRNA hsa‐miR‐199a‐3p expression in interleukin (IL)‐1β‐stimulated human OA chondrocytes. This negative co‐regulation of hsa‐miR‐199a‐3p and COX‐2 by EGCG was confirmed by transfection of OA chondrocytes with anti‐miR‐199a‐3p. Transfection of OA chondrocytes with anti‐miR‐199a‐3p significantly enhanced COX‐2 expression and PGE_2_ production (*P* < 0.001), while EGCG treatment significantly inhibited anti‐miR‐199a‐3p transfection‐induced COX‐2 expression or PGE_2_ production in a dose‐dependent manner. These results were further re‐validated by co‐treatment of these transfection OA chondrocytes with IL‐1β and EGCG. EGCG treatment consistently up‐regulated the IL‐1β‐decreased hsa‐miR‐199a‐3p expression (*P* < 0.05) and significantly inhibited the IL‐1β‐induced COX‐2 expression/PGE_2_ production (*P* < 0.05) in OA chondrocytes transfected with anti‐hsa‐miR‐199a‐3p. Taken together, these results clearly indicate that EGCG inhibits COX‐2 expression/PGE_2_ production *via* up‐regulation of hsa‐miR‐199a‐3p expression. These novel pharmacological actions of EGCG on IL‐1β‐stimulated human OA chondrocytes provide new suggestions that EGCG or EGCG‐derived compounds inhibit cartilage breakdown or pain by up‐regulating the expression of microRNAs in human chondrocytes.

## Introduction

MicroRNAs (miRNAs) are a class of small, non‐coding RNAs that regulate mRNA expression at the post‐transcriptional level. It is assumed that miRNAs regulate ~60% of all protein coding genes in humans and participate in the regulation of almost every cellular events investigated to date 1, 2. The importance of miRNAs in maintaining cartilage homoeostasis during development and their dysregulated expression have recently linked with joint pathologies 2, 3. Dicer is an important component for biogenesis of miRNAs was found to have an essential function in skeletal development. In our previous report, we showed that human chondrocytes express Dicer‐1 transcript and protein 4. These findings confirmed that proteins associated with the biogenesis of miRNAs are expressed in human chondrocytes 2, 4. Now, it becomes well clear that miRNAs play an important role in the onset/progression of osteoarthritis (OA) 2, 3, 4. Osteoarthritis is the most common musculoskeletal disorder leading to significant morbidity because of joint pain and disability. The underlying disease process is thought to be multifactorial and its aetiology remains to be fully investigated 2, 5. Molecular evidence clearly suggest that OA onset is associated with excessive production of interleukin (IL)‐1β, which plays a key role in joints damage *via* up‐regulation of the expression of other inflammatory mediators such as matrix metalloproteinase (MMP)s, cyclooxygenase‐2 (COX‐2), *etc*. 4, 6, 7. Excess induction of COX‐2 expression, leading to the elevated production of prostaglandin E2 (PGE_2_) 8. Although very low levels of PGE_2_ produced by OA tissues are predominantly catabolic, leading to an inhibition of proteoglycan synthesis, increased production of MMP‐13, this further enhanced the degradation of type II collagen in OA joints 8, 9, 10. Therefore, PGE_2_ is regarded as a possible therapeutic target for OA treatment. The main intermediate enzyme responsible for PGE_2_ biosynthesis is COX‐2, which has now becomes the most important target for OA therapy. COX‐2‐selective inhibitors have efficacy in OA that is similar to that of nonsteroidal anti‐inflammatory drugs (NSAIDs) but with a lower potential for upper gastrointestinal injury, a serious side‐effect of nonselective NSAIDs 8, 9, 10, 11. For these reasons, COX‐2 is an obvious target of OA therapy.

Epigallocatechin‐3‐*O‐*gallate (EGCG) is a bioactive polyphenol of green tea, has gained significant attention among scientists and has now become one of the leading naturally derived polyphenols studied for its potential health benefits 12, 13. A cup of green tea typically provides 60–125 mg catechins, including EGCG. EGCG has been shown to be 25–100 times more effective than vitamins C and E in terms of antioxidant activity 13. Haqqi *et al*. performed extensive studies in the past decade and have verified the cartilage‐preserving and chondro‐protective action of EGCG 14, 15, 16, 17. We also have earlier shown that EGCG was non‐toxic to human chondrocytes and inhibited the expression of inflammatory mediator's *in vitro* 18. Recently, several studies have revealed that dietary polyphenols including EGCG have the potential to modulate miRNAs expression in various cancer cells 19, 20, 21, 22, 23. However, the effects of EGCG on miRNA expression in chondrocytes are unknown.

Here, we have addressed the question for the first time of a possible regulatory effect of EGCG on miRNAs regulation in human chondrocytes. In this study, we determined that EGCG inhibits the IL‐1β‐induced COX‐2 mRNA/protein expression *via* up‐regulating the expression of microRNA hsa‐miR‐199a‐3p in primary human OA chondrocytes. Our results thus identify a unique mechanism of action of a dietary constituent of green tea and suggest that use of EGCG or compounds derived from it may have cartilage sparing effect by miRNAs regulation in arthritis.

## Materials and methods

### Patients cartilage and preparation of chondrocytes

Present study has been carried out in accordance with the Code of Ethics of the World Medical Association (Declaration of Helsinki as revised in Tokyo 2004) for humans and was approved by local ethical committee of College of Medicine, Qassim University and King Fahd Medical City, KSA. With Institutional Review Board approvals, discarded cartilage samples were obtained from the knee joints of OA patients (*n* = 12) undergoing joint replacement surgery. The macroscopic cartilage degeneration was determined by staining of femoral head samples with India ink and the cartilage with smooth articular surface was resected and used to prepare chondrocytes by enzymatic digestion as described previously 24. Isolated human chondrocytes (1.2 × 10^6^ million) were plated in 35 mm plates in complete DMEM and incubated for 72 hrs at 37°C with 5% CO_2_ as previously described 25.

### Treatment of primary human chondrocytes with IL‐1β and EGCG

Human OA chondrocytes (1.2 × 10^6^/ml) were plated in complete DMEM medium (catalog # SLM‐120‐B; Millipore Corporation, Temecula, CA, USA) and serum‐starved for 12 hrs/overnight. Starved OA chondrocytes were pre‐treated with different doses of EGCG (purity ≥95%; Calbiochem, San Diego, CA, USA) for 2 hrs prior to stimulation with IL‐1β (5 ng/ml; catalog # IL038; EMD Millipore Corporation) for 8 or 24 hrs as described previously 26. Human OA chondrocytes cultured without IL‐1β or EGCG served as controls.

### Transfection of chondrocytes with miRNA inhibitors

Human OA chondrocytes were transfected with anti‐miRNAs (50 nM; Ambion, Foster City, CA, USA or Qiagen, Hilden, Germany) at a 50 nM concentration, using the calcium phosphate precipitation method 27. Following transfection, chondrocytes were pre‐treated with EGCG (20–50 μM) and then stimulated with IL‐1β (5 ng/ml) for 8–24 hrs to analyse the expression of miRNA, mRNA or protein.

### Preparation of microRNAs, reverse transcription and TaqMan assays

Total RNA containing miRNA fractions was prepared using mirVana miRNA isolation kit (catalog # AM1560; Ambion, Foster City, CA, USA) according to the manufacturers' instructions. Total RNA (0.6 μg) was reverse‐transcribed using SuperScript First Strand cDNA synthesis kit (Applied Biosystems, Foster City, CA, USA). The expression of COX‐2 mRNA and hsa‐miR‐199a‐3p was quantified by TaqMan Gene Expression Assays (Applied Biosystems). Real‐time PCR amplification and data capture were carried out using the Step One Real Time PCR System (Applied Biosystems). GAPDH/RNU6B expression was used as an endogenous control. Relative expression levels were analysed using ΔΔCT method 28.

### Western blotting

Expression of proteins in OA chondrocytes were determined by western immunoblotting as described previously 29. Total cell lysates were prepared using the Pierce RIPA buffer (catalog # 89901; Thermo Scientific, Vernon Hills, IL, USA). Total cell lysates (30 μg/lane) were resolved by SDS‐PAGE (10% resolving gel with 4% stacking) and transferred to nitrocellulose membranes (Bio‐Rad, Hercules, CA, USA). Membranes were blocked with non‐fat dry milk powder in Tris buffered saline and 0.1% Tween‐20 (TBS‐T). Blots were probed with diluted (1:1000) primary antibodies specific for the COX‐2 (catalog # D5H5; Cell Signaling Technology, Beverley, MA, USA) and β‐actin (catalog # 8457; Cell Signaling Technology). Immunoreactive proteins were visualized by using 1:1000 diluted HRP‐linked secondary antibodies and enhanced chemiluminescence (GE Healthcare, Milwaukee, WI, USA). Images were analysed using the UN‐SCAN‐IT (Silk Scientific Corporation, Orem, UT, USA). Each band was scanned five times with background correction and values were expressed as average pixel band ratios.

### Prostaglandin E_2_ ELISA

Prostaglandin E_2_ production in the culture medium of treated or untreated human OA chondrocytes was quantified using commercially available ELISA kit (Cayman Chemicals, Ann Arbor, MI, USA) according to the instructions of the manufacturer. ELISA plate was read using an automatic microplate reader (Anthos Zenyth 3100 Multimode Detectors, Salzburg, Austria).

### Statistical analysis

Statistical comparisons were performed by one‐way anova analysis followed by Tukey's post‐hoc analysis or Two‐way anova followed by Bonferroni post‐hoc tests using Graph Pad Prism‐5 (San Diego, CA, USA) and *P* < 0.05 was considered significant.

## Results

### Primary chondrocytes maintain their chondrogenic behaviour

We first determined whether primary chondrocytes used in this study maintained their phenotypic behaviours. Our results show that primary chondrocytes maintained their behaviours, when they were plated (1.2 × 10^6^/ml) in 35 mm culture dishes, as judged by the continued expression of Col2A1, ACAN and SOX‐9 mRNAs (data not shown). Based on this data, chondrocytes were used within 72 hrs after plating to avoid de‐differentiation of human OA chondrocytes.

### EGCG up‐regulates hsa‐miR‐199a‐3p expression

**Figure 1:**
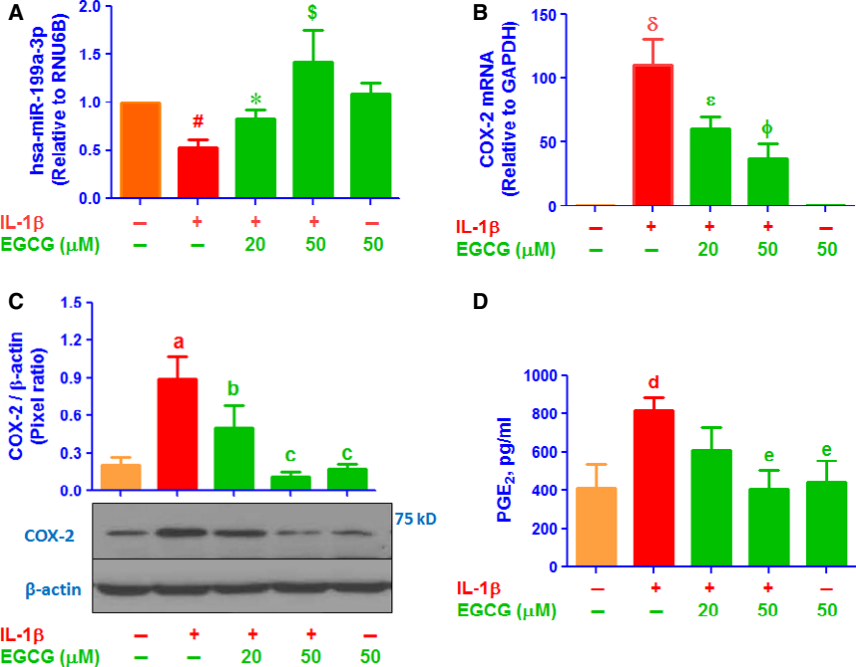
EGCG up‐regulates hsa‐miR‐199a‐3p expression and down‐regulates COX‐2 expression in IL‐1β‐stimulated human OA chondrocytes. (**A**) Effect of EGCG on IL‐1β‐induced down‐regulation of hsa‐miR‐199a‐3p in human OA chondrocytes determined by TaqMan assays. ^#^ *P* < 0.05 *versus* control; *P < 0.05 *versus* #; ^$^ *P* < 0.01 *versus* #. (**B**) Effect of EGCG on IL‐1β‐induced up‐regulation of COX‐2 mRNA expression determined by TaqMan assay. ^δ^ *P* < 0.0001 *versus* control; ^ε^ *P* < 0.05 *versus* δ; ^ϕ^ *P* < 0.001 *versus* δ. (**C**) Effect of EGCG on IL‐1β‐induced up‐regulation of COX‐2 protein expression determined COX‐2 protein expression determined by western blotting. ^a^ *P* < 0.0001 *versus* control; ^b^ *P* < 0.01 *versus* a; ^c^ *P* < 0.001 *versus* a. (**D**) Effect of EGCG on IL‐1β‐induced PGE_2_ production in the culture medium of human OA chondrocytes. ^d^ *P* < 0.01 *versus* control; ^e^ *P* < 0.01 *versus* d. Unstimulated chondrocytes were used as controls and expression of RNU6B/GAPDH was used as an endogenous control.

Primary OA chondrocytes (70–80% confluent) were pre‐treated with EGCG (20–50 μM) for 2 hrs, then stimulated with IL‐1β (5 ng/ml) for 8–24 hrs. No cytotoxic effect of EGCG and IL‐1β was noted at the dose used (results not shown). The expression level of mature hsa‐miR‐199a‐3p was quantified by TaqMan Small RNA Assays (Applied Biosystems) using quantitative RT‐PCR method, and values were compared with control. Stimulation of human OA primary chondrocytes with IL‐1β alone showed a significant down‐regulation of hsa‐miR‐199a‐3p expression (*P* < 0.05). Interestingly, treatment of OA chondrocytes with EGCG significantly enhanced IL‐1β‐induced hsa‐miR‐199a‐3p expression in a dose‐dependent manner (Fig. 1A; *P* < 0.05). However, EGCG alone had no significant effect on hsa‐miR‐199a‐3p expression (*P* > 0.05). In addition, we also determined the COX‐2 mRNA expression, Figure 1B shows that human OA chondrocytes treated with IL‐1β alone had higher level of COX‐2 mRNA compared with untreated OA chondrocytes (*P* < 0.001). However, pretreatment of OA chondrocytes with EGCG showed marked decline of IL‐1β‐induced COX‐2 mRNA (*P* < 0.05). To determine whether inhibition of COX‐2 mRNA expression also affected COX‐2 protein, chondrocytes lysates were prepared and assayed for COX‐2 protein. As shown in Figure 1C, pretreatment with EGCG significantly suppressed the IL‐1β‐induced COX‐2 protein and the maximum suppression was observed at 50 μM of EGCG. As expected, treatment of OA chondrocytes with EGCG alone showed no effect on COX‐2 mRNA and protein expression (Fig. 1B and C; *P* > 0.05). Not only these, we also investigated the effect of EGCG on the production of PGE_2_. The levels of PGE_2_ in the culture medium were estimated by PGE_2_‐specific ELISA. Our analysis revealed that stimulation of chondrocytes with IL‐1β alone produced more PGE_2_ as compared to unstimulated OA chondrocytes (*P* < 0.001). However, EGCG alone had no significant effect on PGE_2_ production in the culture medium of OA chondrocytes (*P* > 0.05). Importantly, IL‐1β‐induced PGE_2_ production was significantly inhibited by EGCG in a dose‐dependent manner (Fig. 1D). Inhibition of IL‐1β‐induced PGE_2_ production by EGCG may have correlation with the up‐regulation of hsa‐miR‐199a‐3p expression and down‐regulation of COX‐2 gene in human OA chondrocytes.

### Negative co‐regulation of hsa‐miR‐199a‐3p and COX‐2 by EGCG

**Figure 2:**
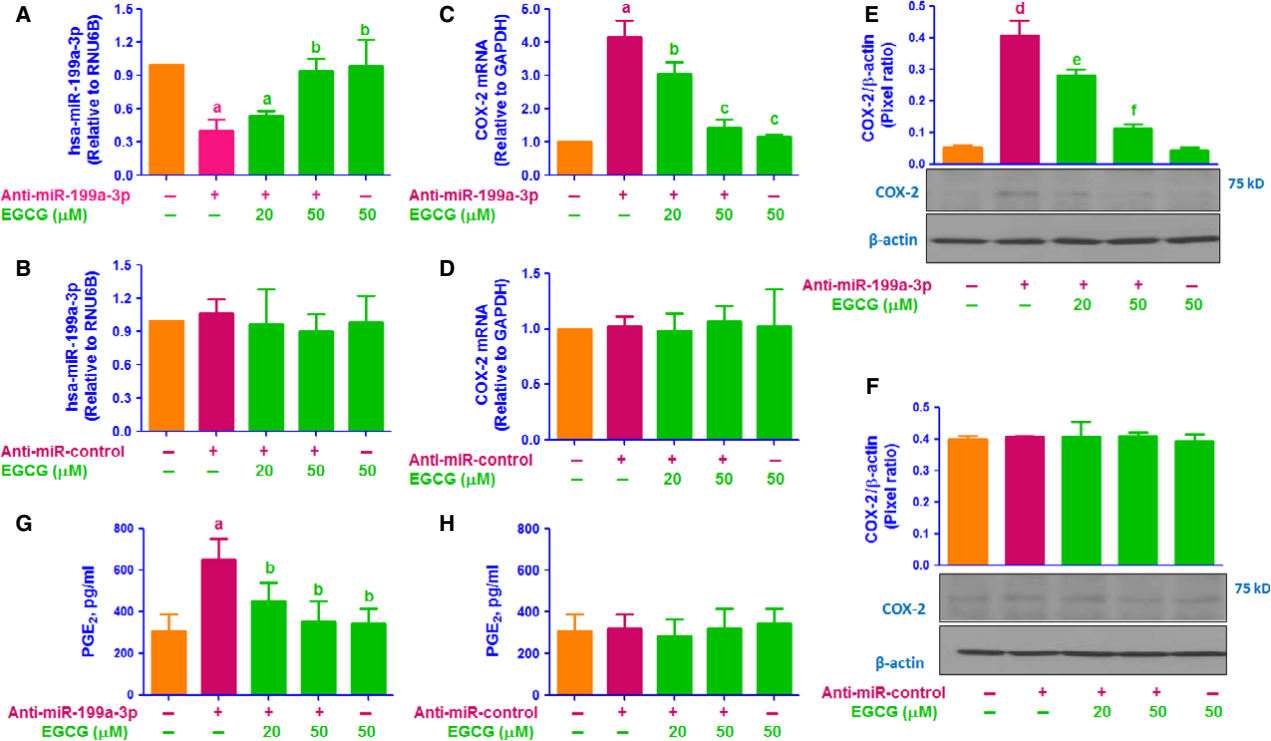
EGCG up‐regulates the hsa‐miR‐199a‐3p expression, down‐regulates COX‐2 expression and PGE_2_ production in anti‐miR‐199a‐3p‐transfected OA chondrocytes. (**A**) Effect of EGCG on hsa‐miR‐199a‐3p expression in human OA chondrocytes transfected with anti‐miR‐199a‐3p. ^a^ *P* < 0.01 *versus* control; ^b^ *P* < 0.05 *versus* a. (**B**) Effect of EGCG on hsa‐miR‐199a‐3p expression in human OA chondrocytes transfected with anti‐miR‐control. (**C**) Effect of EGCG on COX‐2 mRNA expression in human OA chondrocytes transfected with anti‐miR‐199a‐3p. ^a^ *P* < 0.001 *versus* control; ^b^ *P* < 0.01 *versus* a; ^b^ *P* < 0.05 *versus* c. (**D**) Effect of EGCG on COX‐2 mRNA expression in human OA chondrocytes transfected with anti‐miR‐control. (**E**) Effect of EGCG on COX‐2 protein expression in human OA chondrocytes transfected with anti‐miR‐199a‐3p. ^d^ *P* < 0.01 *versus* control; ^d^ *P* < 0.05 *versus* e; ^e^ *P* < 0.001 *versus* f. (**F**) Effect of EGCG on COX‐2 protein expression in human OA chondrocytes transfected with anti‐miR‐control. (**G**) Effect of EGCG on PGE_2_ production in the culture medium of human OA chondrocytes transfected with anti‐miR‐199a‐3p. ^a^ *P* < 0.001 *versus* control; ^a^ *P* < 0.01 *versus* b. (**H**) Effect of EGCG on PGE_2_ production in the culture medium of human OA chondrocytes transfected with anti‐miR‐control. Unstimulated chondrocytes were used as controls and expression of RNU6B, GAPDH or β‐action were used as an endogenous control.

**Figure 3:**
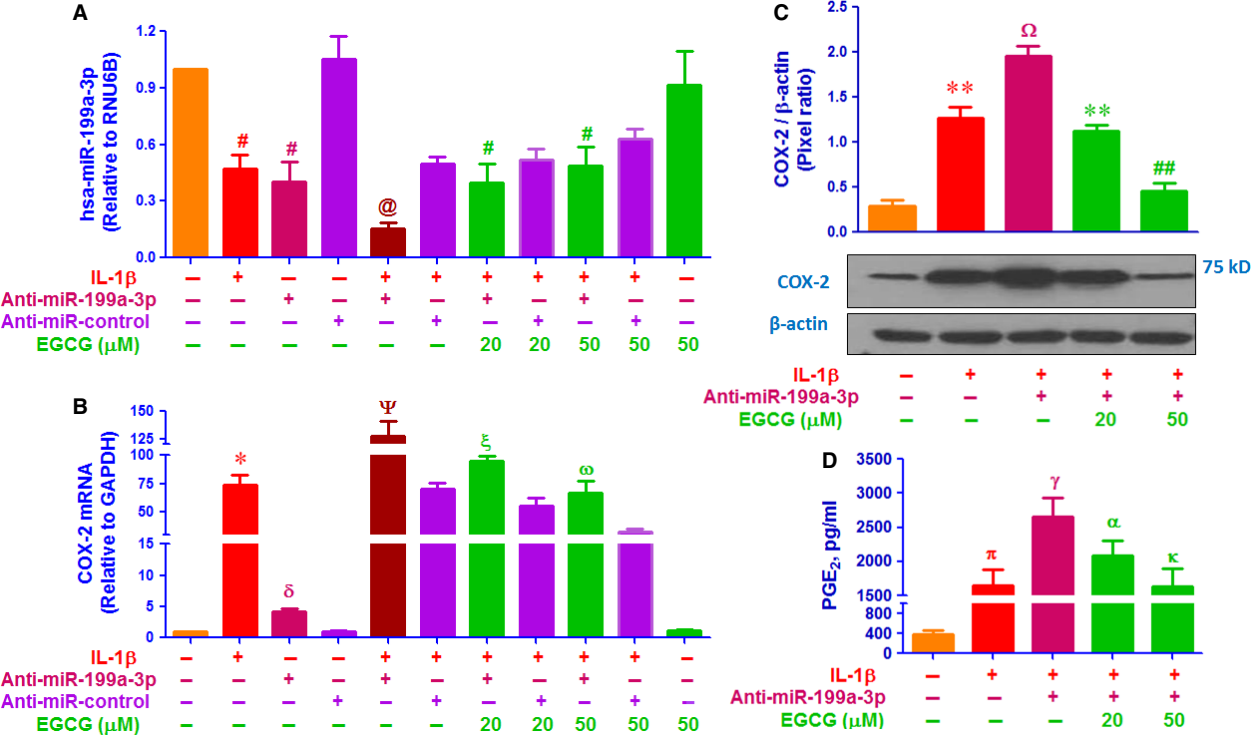
EGCG up‐regulates the IL‐1β‐decreased hsa‐miR‐199a‐3p expression and down‐regulates the IL‐1β‐induced COX‐2 expression in anti‐miR‐199a‐3p‐transfected OA chondrocytes. (**A**) Effect of EGCG on IL‐1β‐decreased hsa‐miR‐199a‐3p expression in human OA chondrocytes transfected with anti‐miR‐199a‐3p. ^#^ *P* < 0.01 *versus* control; ^@^ *P* < 0.05 *versus* chondrocytes transfected with anti‐miR‐control alone; ^@^ *P* < 0.05 *versus* #. (**B**) Effect of EGCG on IL‐1β‐induced COX‐2 mRNA expression in human OA chondrocytes transfected with anti‐miR‐199a‐3p. *P < 0.0001 *versus* control; ^δ^ *P* < 0.05 *versus* chondrocytes transfected with anti‐miR‐control alone; ^Ψ^ *P* < 0.05 *versus* ξ; ^ξ^ *P* < 0.05 *versus* ω. (**C**) Effect of EGCG on IL‐1β‐induced COX‐2 protein expression in human OA chondrocytes transfected with anti‐miR‐199a‐3p. **P < 0.01 *versus* control; **P < 0.05 *versus* Ω; **P < 0.05 *versus* ##. (**D**) Effect of EGCG on IL‐1β‐induced PGE_2_ production in the culture medium of human OA chondrocytes transfected with anti‐miR‐199a‐3p. ^π^ *P* < 0.001 *versus* control; ^γ^ *P* < 0.01 *versus* π; ^α^ *P* < 0.05 *versus* α; ^α^ *P* < 0.01 *versus* κ. Unstimulated chondrocytes were used as controls and expression of RNU6B, GAPDH and β‐actin were used as endogenous controls.

Involvement of EGCG in the negative co‐regulation of hsa‐miR‐199a‐3p and COX‐2 was confirmed by transfection of human OA chondrocytes with anti‐miR‐199a‐3p used as miRNA inhibitor. In these studies, human OA chondrocytes were transfected with anti‐miR‐199a‐3p or anti‐miR‐control (50 nM) and were then treated with EGCG (20–50 μM). Transfection of OA chondrocytes with anti‐miR‐199a‐3p decreased the hsa‐miR‐199a‐3p expression compared to control chondrocytes (*P* < 0.001). However, treatment of these transfected OA chondrocytes with EGCG significantly enhanced hsa‐miR‐199a‐3p expression in a dose‐dependent manner (Fig. 2A; *P* < 0.05). In the same experimental settings, human OA chondrocytes were also transfected with anti‐miR‐control, followed by EGCG (20–50 μM). Transfection of OA chondrocytes with negative control anti‐miR‐control had no significant effect on hsa‐miR‐199a‐3p expression compared to control chondrocytes (*P* > 0.05). As expected, EGCG treatment also had no effect on hsa‐miR‐199a‐3p expression in OA chondrocytes transfected with anti‐miR‐control (Fig. 2B; *P* > 0.05). We determine whether up‐regulation of hsa‐miR‐199a‐3p by EGCG also modulates COX‐2 expression in anti‐miR‐199a‐3p‐transfected chondrocytes, total RNA were prepared, Figure 2C shows that transfection of OA chondrocytes with anti‐miR‐199a‐3p significantly up‐regulated COX‐2 mRNA expression compared to control chondrocytes (*P* < 0.0001). Interestingly, EGCG significantly inhibited this up‐regulated COX‐2 mRNA in a dose‐dependent manner (*P* < 0.05). Although transfection of OA chondrocytes with anti‐miR‐control, followed by EGCG treatment had no effect on COX‐2 mRNA expression (Fig. 2D; *P* > 0.05). To determine whether these modulations of COX‐2 mRNA expression also affected the protein level, cell lysates were assayed for COX‐2 protein. As shown in Figure 2E, transfection of OA chondrocytes with anti‐miR‐199a‐3p affectedly increased the COX‐2 protein expression (*P* < 0.001). This increase in COX‐2 protein expression was reversed by EGCG treatment in a dose‐dependent manner in anti‐miR‐199a‐3p transfected chondrocytes (Fig. 2E; *P* < 0.05). As expected, transfection of OA chondrocytes with anti‐miR‐control, followed by EGCG treatment had no effect COX‐2 protein expression (Fig. 3F; *P* > 0.05). We also determine whether inhibition of COX‐2 expression also inhibits PGE_2_ production, chondrocytes culture medium were analysed. Transfection of OA chondrocytes with anti‐miR‐199a‐3p affectedly increased the PGE_2_ production (*P* < 0.01). This increase in PGE_2_ production was significantly inhibited by EGCG in a dose‐dependent manner (Fig. 2G; *P* < 0.05). As expected, transfection of OA chondrocytes with anti‐miR‐control, followed by EGCG treatment had no effect on PGE_2_ production (Fig. 2H; *P* > 0.05). Epigallocatechin‐3‐*O*‐gallate effects on the negative co‐regulation of hsa‐miR‐199a‐3p and COX‐2 was further re‐validated by transfection of OA chondrocytes with anti‐miR‐199a‐3p followed by co‐treatment with IL‐1β and EGCG. Transfection of OA chondrocytes with anti‐miR‐199a‐3p synergizes with IL‐1β in reducing miR‐199a‐3p levels (*P* < 0.001) (Fig. 3A; bar 5). Although, EGCG treatment remarkably and consistently up‐regulated the IL‐1β‐inhibited hsa‐miR‐199a‐3p expression in a dose‐dependent manner (Fig. 3A; *P* < 0.05). As expected transfection of OA chondrocytes with anti‐miR‐control without IL‐1β or EGCG had no effect on hsa‐miR‐199a‐3p expression, but IL‐1β significantly down‐regulated hsa‐miR‐199a‐3p expression (*P* < 0.05) and this IL‐1β‐induced down‐regulation of hsa‐miR‐199a‐3p expression was slightly up‐regulated by EGCG (Fig. 3A; *P* > 0.05). In the identical experimental conditions, COX‐2 mRNA expression was also measured, Figure 3B shows that OA chondrocytes treated with IL‐1β alone had a higher level of COX‐2 mRNA compared to untreated OA chondrocytes (*P* < 0.001). Transfection of OA chondrocytes with anti‐miR‐199a‐3p showed marked increased of IL‐1β‐induced COX‐2 mRNA expression as compared with IL‐1β‐treated chondrocytes transfected with anti‐miR‐control (Fig. 3B; *P* < 0.05). Interestingly, EGCG significantly inhibited IL‐1β‐induced COX‐2 mRNA in a dose‐dependent manner in OA chondrocytes transfected with anti‐miR‐199a‐3p (Fig. 3B; *P* < 0.05). As expected transfection of OA chondrocytes with anti‐miR‐control without IL‐1β or EGCG had no effect on COX‐2 mRNA expression, but IL‐1β significantly up‐regulated COX‐2 mRNA (*P* < 0.05) and this IL‐1β‐induced COX‐2 mRNA expression was slightly inhibited by EGCG (Fig. 3B; *P* > 0.05). To determine whether these modulations of COX‐2 mRNA expression also affected the protein level, cell lysates were assayed for COX‐2 protein. Analysis of cell lysates for COX‐2 protein showed that transfection of OA chondrocytes with anti‐miR‐199a‐3p effectively further enhanced the IL‐1β‐induced COX‐2 protein expression (*P* < 0.01). This enhanced expression of COX‐2 protein was significantly inhibited by EGCG in a dose‐dependent manner (Fig. 3C; *P* < 0.05). Taken together, the data clearly indicate has‐miR‐199a‐3p is a direct regulator of COX‐2 expression in human OA chondrocytes and EGCG induced inhibition of COX‐2 mRNA/protein expression is mediated *via* up‐regulation of hsa‐miR‐199a‐3p expression. Overexpression of COX‐2, a key mediator of inflammation, and its product, PGE_2_ are associated with cartilage degradation and pain in OA. We determine whether inhibition of COX‐2 expression also inhibits PGE_2_ production, chondrocytes culture medium were analysed. Transfection of OA chondrocytes with anti‐miR‐199a‐3p transfection effectively further increased the IL‐1β‐induced PGE_2_ (*P* < 0.01) in OA chondrocytes. This enhanced expression of PGE_2_ production was consistently inhibited by EGCG in a dose‐dependent manner (Fig. 3D; *P* < 0.05). Taken together, these results clearly indicate that EGCG inhibits COX‐2 expression/PGE_2_ production *via* up‐regulation of hsa‐miR‐199a‐3p expression. These results are novel and have not been previously reported.

## Discussion

This is the first report that shows green tea polyphenol EGCG inhibits COX‐2 expression by up‐regulating the expression of microRNA hsa‐miR‐199a‐3p in human chondrocytes. Research on the role of miRNAs in OA has been very active for the past 5 years. Many novel miRNAs have been discovered that regulate the expression of key genes relevant to OA pathogenesis. Recently, we reported that microRNA hsa‐miR‐26a‐5p regulates the expression of inducible nitric oxide synthase in human OA chondrocytes 30. In another study, we also reported that hsa‐miR‐27b regulates the expression of MMP‐13 in human OA chondrocytes 4. Haqqi *et al*. demonstrated that miR‐199a* (previous ID of hsa‐miR‐199a‐3p) regulates COX‐2 expression in IL‐1β‐stimulated human chondrocytes 31. Moreover, Park *et al*. showed that hsa‐miR‐558 also regulates IL‐1β‐induced COX‐2 expression in human chondrocytes 32. Now it is well‐established that miRNAs play significant roles in all major aspects of cartilage development, homoeostasis and pathology 1, 2, 3. Persistence of IL‐1β in OA joints is an important characteristic of OA pathology, which is produced by inflamed synovium and affected OA chondrocytes 6, 7. Human chondrocytes are highly responsive to IL‐1β and the most striking effect of IL‐1β on chondrocytes is to induce COX‐2 expression and PGE_2_ production, which is potent source for inflammatory 7, 8, 9, 10. Although arthritis is present in every population and OA is the most common joint disorder but the treatment is still limited to a few classes of drugs, primarily NSADs and injectable corticosteroids. However, while providing relief from pain, none of these drugs has been shown to inhibit disease progression. They also have varying degrees of gastrointestinal toxicity and cardiovascular risk 33. Therefore, there is a need for the development or identification of new compounds that inhibit the catabolic process in OA joints and are better to be tolerated by the gastrointestinal tract than currently available anti‐OA drugs. Previously, we have shown that EGCG inhibits the production of MMP‐13 and tumour necrosis factor‐α in stimulated OA chondrocytes 18 and now EGCG has gained significant attention among scientists and has been one of the leading naturally occurring molecules studied for its potential benefits for arthritis patients 12, 13, 14, 15, 16, 17. Recently, the regulation of miRNAs by natural, non‐toxic agents (including EGCG) has been demonstrated. By conducting *in vivo* studies, diets with natural occurring agents in animals were found to regulate the expression of miRNAs 19. Moreover, treatment of human hepatic cancer cells with EGCG inhibits the expression of anti‐apoptotic protein Bcl‐2 by up‐regulating the expression of miRNA miR‐16 20. Similarly, EGCG also up‐regulates the expression of miR‐1 and down‐regulates the expression of c‐MET in MG‐63 and U‐2OS cells 21. EGCG treatment also promotes apoptosis of U2OS cells *via* up‐regulating the expression of miR‐126 22. In addition, EGCG induced up‐regulation of miRNA let‐7b expression led to down‐regulation of high mobility group A2, a target gene related to tumour progression 23.

This study is the first to determine the role of EGCG in silencing the effect of pro‐inflammatory genes *via* regulation of miRNA in human chondrocytes. Our results show the effect of EGCG on miRNA‐mediated post‐transcriptional alterations of COX‐2 expression in human OA chondrocytes. Haqqi *et al*. were the first to identify seed‐matched sequence of 3′UTR of COX‐2 mRNA with hsa‐miR‐199a‐3p by Target Scan algorithm 31. They also showed COX‐2 expression was regulated directly by hsa‐miR‐199a‐3p in IL‐1β‐stimulated human chondrocytes 31. Here, we demonstrated that treatment of OA chondrocytes with EGCG significantly enhanced hsa‐miR‐199a‐3p expression in IL‐1β‐stimulated human OA chondrocytes. Moreover, our results also show that EGCG induced marked decline of COX‐2 gene and protein expression. These findings indicate that EGCG inhibits COX‐2 expression by up‐regulating the expression of hsa‐miR‐199a‐3p. Involvement of EGCG in this negative co‐regulation of hsa‐miR‐199a‐3p and COX‐2 was confirmed by transfection of human OA chondrocytes with anti‐miR‐199a‐3p. Treatment of transfected chondrocytes with EGCG remarkably up‐regulated the IL‐1β‐inhibited hsa‐miR‐199a‐3p expression and markedly inhibited the IL‐1β‐induced COX‐2 mRNA/protein expression. These findings clearly indicate that EGCG inhibits COX‐2 expression *via* up‐regulation of hsa‐miR‐199a‐3p expression.

Human chondrocytes are thought to be the major source of PGE_2_ secretion in OA joints and inhibition of COX‐2 expression and PGE_2_ secretion was found to be chondroprotective 8, 9, 10, 29. Our results also showed that even without stimulant OA chondrocytes produced PGE_2_ but the levels becomes significantly enhanced upon IL‐1β treatment (*P* < 0.05). Here, we also determine whether inhibition of COX‐2 expression by EGCG also inhibits PGE_2_ production. Transfection of OA chondrocytes with anti‐miR‐199a‐3p effectively further increased the IL‐1β‐induced PGE_2_ production. This increased of PGE_2_ production was consistently inhibited by EGCG. Taken together, the data clearly indicate that EGCG inhibits PGE_2_ production *via* negative regulation of COX‐2 and hsa‐miR‐199a‐3p expression. These novel results may indicate that EGCG inhibits cartilage breakdown and pain *via* up‐regulation of microRNA hsa‐miR‐199a‐3p expression, which can be exploited in a newer therapeutic approach for the treatment of OA and other degenerative/inflammatory disorders.

## Conclusions

The present article is the first report that shows green tea catechin EGCG inhibits the inflammatory activity by modulating the expression of microRNA in human chondrocytes. Our results indicate that EGCG inhibits IL‐1β‐induced COX‐2 expression or PGE_2_ production *via* up‐regulation of the expression of microRNA hsa‐miR‐199a‐3p in human OA chondrocytes. These novel pharmacological actions of EGCG on microRNA regulation provide new suggestions that EGCG or EGCG‐derived compounds may be of value for the treatment of inflammatory arthritis in which microRNAs play an active role.

## Disclosure

The authors declare no conflict of interest.

## Author contribution

All authors carried out experimentation, data interpretation and manuscript drafting. ZR conceived of the study, its design, coordination, data interpretation and manuscript drafting. All authors have read and approved the final manuscript.
